# A combination approach of pseudotime analysis and mathematical modeling for understanding drug-resistant mechanisms

**DOI:** 10.1038/s41598-021-97887-z

**Published:** 2021-09-16

**Authors:** Shigeyuki Magi, Sewon Ki, Masao Ukai, Elisa Domínguez-Hüttinger, Atsuhiko T Naito, Yutaka Suzuki, Mariko Okada

**Affiliations:** 1 Laboratory of Cell Systems, Institute for Protein Research, Osaka University, Osaka, 565-0871 Japan; 2 Laboratory for Integrated Cellular Systems, RIKEN Center for Integrative Medical Sciences (IMS), Yokohama, 230-0045 Japan; 3 Department of Physiology, Division of Cell Physiology, Faculty of Medicine, Toho University, Tokyo, 143-8540 Japan; 4 Graduate School of Medical Life Science, Yokohama City University, Yokohama, 230-0045 Japan; 5 Departamento de Biología Molecular y Biotecnología, Instituto de Investigaciones Biomédicas, Universidad Nacional Autónoma de México, Ciudad Universitaria, 04510 México, México; 6 Department of Computational Biology and Medical Sciences, Graduate School of Frontier Sciences, The University of Tokyo, Chiba, 277-8562 Japan; 7 Center for Drug Design and Research, National Institutes of Biomedical Innovation, Health and Nutrition, Ibaraki, Osaka 567-0085 Japan

**Keywords:** Computational biology and bioinformatics, Breast cancer

## Abstract

Cancer cells acquire drug resistance through the following stages: nonresistant, pre-resistant, and resistant. Although the molecular mechanism of drug resistance is well investigated, the process of drug resistance acquisition remains largely unknown. Here we elucidate the molecular mechanisms underlying the process of drug resistance acquisition by sequential analysis of gene expression patterns in tamoxifen-treated breast cancer cells. Single-cell RNA-sequencing indicates that tamoxifen-resistant cells can be subgrouped into two, one showing altered gene expression related to metabolic regulation and another showing high expression levels of adhesion-related molecules and histone-modifying enzymes. Pseudotime analysis showed a cell transition trajectory to the two resistant subgroups that stem from a shared pre-resistant state. An ordinary differential equation model based on the trajectory fitted well with the experimental results of cell growth. Based on the established model, it was predicted and experimentally validated that inhibition of transition to both resistant subtypes would prevent the appearance of tamoxifen resistance.

## Introduction

Estrogen receptor (ER) is a hormone-dependent transcription factor that plays an important role in many physiological processes, including reproductive development, bone homeostasis, and cardiovascular remodeling. ER is also closely associated with breast cancer development^1,2^. Approximately 75% of all breast cancer cases are categorized into ER-positive luminal subtypes^3^ and initially treated using an ER antagonist, tamoxifen (TAM). Unfortunately, around 40% of TAM-responsive tumors progress to resistant and metastatic tumors after long-term treatment^4^. The molecular mechanisms by which those tumors exhibit TAM resistance, have been shown to involve alterations in the estrogen–ER interaction-dependent gene expression^5^, cholesterol pathway^6^, and histone demethylase activity (which regulates cellular transcriptomic heterogeneity)^7^ resulting in hyperactivation of alternative signaling pathways including ErbB receptors^8,9^, ERK1/2^10^, PI3K^11–13^, and NF-κB signaling^14^. However, little is known about the dynamic process of TAM resistance acquisition, i.e., when or how the factors involved in resistance acquisition are altered, because most of the previous studies focused on the end-point comparison between resistant and nonresistant states.

Previously, we analyzed the changes in gene expression of TAM-treated MCF-7 cells, a human ER-positive breast cancer cell line, by bulk RNA-sequencing (RNA-seq) and reported several molecular changes that preceded full acquisition of TAM resistance^15^. However, our analysis might have overlooked the involvement of intracellular heterogeneity in the process of TAM resistance acquisition. In luminal breast cancer patients, single-cell transcriptome and epigenomic surveys revealed that non-genomic cell-to-cell variability generates phenotypic heterogeneity^16^. Therefore, understanding the process of TAM resistance acquisition at a single-cell resolution may be important to fully understand the process of TAM resistance acquisition and develop strategies for preventing cancer recurrence.

In this study, we sequentially analyzed transcriptomic profiles in MCF-7 cells treated with TAM by single-cell RNA-seq. We report that TAM-resistant cells can be subgrouped into two subgroups, one showing altered gene expression related to metabolic regulation and the other showing high expression of genes encoding adhesion molecules and histone-modifying enzymes. Pseudotime analysis of single-cell RNA-seq data revealed a cell transition trajectory to the two resistant subpopulations that stem from a shared pre-resistant state. An ordinary differential equation model based on the cell trajectory fitted well with the experimental results of cell growth. Finally, we experimantally validated our model prediction that the conbinatorial inhibition of two important molecules for each resistant subgroup could repress the growth of resistant cells.

## Results

### Time-series transcriptome profiles of MCF-7 cells during continuous TAM treatment

**Figure 1:**
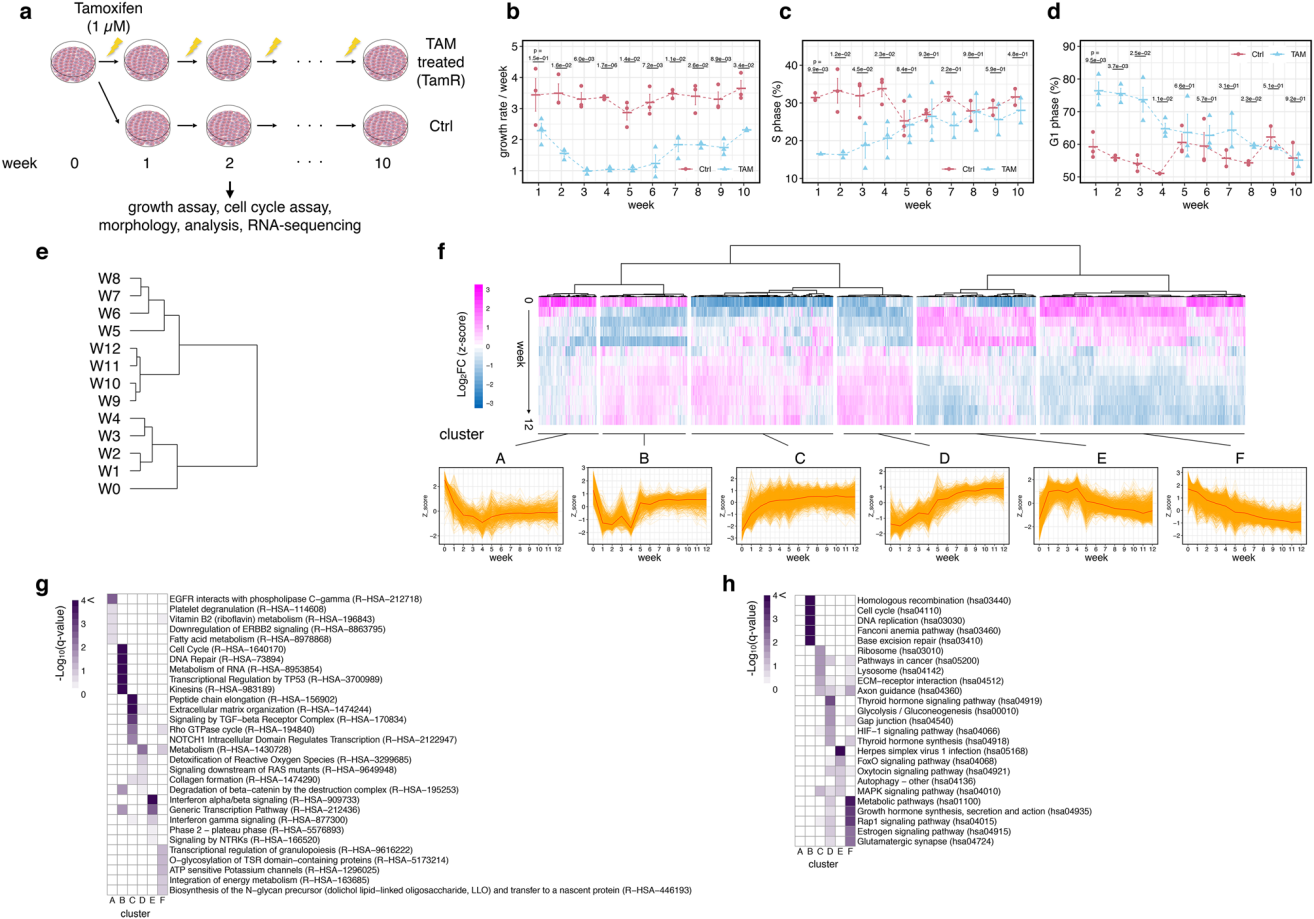
Functional analysis of gene expression patterns in human breast adenocarcinoma MCF-7 cells during the TAM resistance acquisition process. (**a**) Schematic overview of the experimental procedure. Each treatment was replicated five times. (**b**) Growth rate of TAM-treated and control (Ctrl) cells. Data represent mean ± standard error (SE, n = 3). (**c**,**d**) Ratio of S (**c**) and G1 (**d**) phase in TAM-treated and Ctrl cells. Data represent mean ± SE (n = 3 in week 1 to week 6, n = 2 in week 7 to week10). From (**b**) to (**d**), the data points are slightly shifted in the x-axis direction to improve the visibility and prevent overlapping of the Ctrl and TAM graphs, and p-values were calculated using two-tailed Welch's test. (**e**,**f**) Cluster analysis of the z-scores of log2 fold change (log2FC) values by time points (**e**) and by genes (**f**). The bottom line graphs in (**f**) showed individual (orange) or median (red) of gene expression patterns. (**g**,**h**) Heatmaps of enrichment analysis data. The top five significant terms in the Reactome pathway database (**g**) and KEGG pathway database (**h**) in each cluster are presented.

We first investigated the effect of continuous TAM treatment on human breast cancer MCF-7 cells, whose growth depends on ER signaling (Fig. 1a). Treatment with 1 µM TAM initially inhibited cell growth (Fig. 1b) through decreasing the number of cells in S phase whereas increasing that in G1 phase (Fig. 1c,d), suggesting that TAM treatment induced G1 arrest. The growth of cells was almost completely inhibited until week 5 (W5) but recovered thereafter (Fig. 1b). The cell cycle of TAM-treated cells was also dysregulated until W5 but became comparable to control cells after week 6 (W6) (Fig. 1c,d). These results showed the process by which the cells survived and restored their growth potential in the absence of ER signaling.

We next analyzed the bulk RNA-seq data of TAM-treated MCF-7 cells to identify the difference in the gene regulatory network from parental cells. Previously, we performed time-course bulk RNA-seq analysis of TAM-treated MCF-7 cells and non-treated MCF-7 cells, and identified gene sets that play critical role at the “tipping” point of resistance acquisition^15^. In this study, we re-analyzed the dataset in order to focus on time-dependent changes in the expression of each gene, correcting for the effect of the difference in library preparation processes between samples (“Methods” and Supplementary Fig. S1). A total of approximately 6000 differentially expressed genes (DEGs) were identified between TAM-treated and control cells, of which approximately 3000 were up-regulated (Supplementary Fig. S2).

Gene expression in TAM-treated cells was normalized to control cells at each time point, and log2 fold change (log2FC) values were calculated. The log2FC values of all genes at week 0 were set as a theoretical value of zero. We then obtained time-course patterns of the log2FC values of 6982 DEGs at 13 time points and analyzed the similarity of the log2FC values of all DEGs in each week using cluster analysis (Fig. 1e). The expression patterns of DEGs at each time point were classified into four stages; week 0 (W0), from week 1 (W1) to week 4 (W4), W5 to week 8 (W8), and week 9 (W9) to week 12 (W12). The Pearson’s correlation distance from a previous week was the largest at W5, which preceded the recovery of cell growth (Supplementary Fig. S3). In addition, the distance at W1 to W9 was larger than that at week 10 (W10) to W12, indicating that DEG expression patterns became stable at the later stage.

We then investigated the relationships between dynamic gene expression patterns and gene functions by evaluating the similarity of the time-course patterns among all DEGs. Cluster analysis of the z-score of log2FC values revealed six groups of genes with distinct expression patterns: cluster A, rapidly decreasing expression; cluster B, initially down-regulated and recovered at W5; cluster C, rapidly increasing before cell growth rate recovery; cluster D, a gradual increase in expression concomitant with growth rate recovery; cluster E, initially up-regulated and then down-regulated; and cluster F, gradually decreasing (Fig. 1f and Supplementary Table S1). The enrichment analysis of each group was carried out using the Reactome pathway (Fig. 1g) and KEGG (Fig. 1h) databases^17^. Cluster A (rapidly decreasing expression pattern) was enriched in genes related to both receptor tyrosine kinase signaling and fatty acid metabolism. Genes in cluster C (rapid up-regulation) were related to TGFβ signaling or extracellular matrix–receptor interaction, such as *TGFB2*, *SMAD3*, and *MMP9*, or encoded collagen or laminin proteins. This implies that the reorganization of the gene network regulating epithelial–mesenchymal transition or extracellular matrix secretion, both of which contribute to cancer malignancy, occurred before cell growth recovery in the TAM-treated condition. Genes related to ribosomes were also enriched in cluster C, indicating that ribosomal biogenesis may be up-regulated during continuous TAM treatment. On the other hand, cluster D, in which gene expression level increased gradually, was enriched in genes functioning in the thyroid hormone signaling pathway, HIF1 pathway, and glycolytic process, and downstream signaling of RAS, among others. These data show that a Warburg-like effect co-occurs with TAM resistance acquisition. Genes in cluster B showed a non-monotinic dynamic expression pattern characterized by a transient decline from week 1 to week 4, followed by recovery to the basal level. This trend was similar to the growth rate pattern observed in the TAM-treated condition. Cluster B contained numerous genes involved in cell cycle regulation such as *CCND1* and *E2F1*, and DNA replication such as *RAD51*, DNA damage (DNA repair, transcriptional regulation of TP53, and base excision repair), RNA metabolism, kinesins, beta-catenin degradation, generic transcription pathways, and the Fanconi anemia pathway. Genes in cluster E were up-regulated only when cell growth was effectively inhibited by TAM; this pattern was opposite to that observed in cluster B. Cluster E was enriched in genes involved in interferon signaling, FoxO signaling, autophagy, as well as transcription pathways, exytocin signaling pathways, and the MAPK signaling pathway. The interferon and FoxO signaling pathways exhibit anti-survival functions in cancer cells exposed to anti-cancer agents^18^, whereas autophagy contributes to cell survival under normal conditions^19^, suggesting that genes in cluster E reflect both antitumor as well as adaptation mechanisms triggered by the TAM treatment. Cluster F was the largest dynamic cluster containing 2088 genes and was characterized by a consistent decrease in gene expression. Enrichment analysis showed that cluster F contained genes related to multiple cellular functions including energy metabolism, growth hormone synthesis, Rho GTPase (Rap1) signaling, and crucially, the estrogen signaling pathway, suggesting that TAM treatment inhibits the estrogen-dependent gene expression mechanism, and TAM resistance observed in our experiment may be supported by an estrogen/ER-independent mechanism.

### Single-cell RNA-seq analysis of MCF-7 cells under continuous TAM treatment

**Figure 2:**
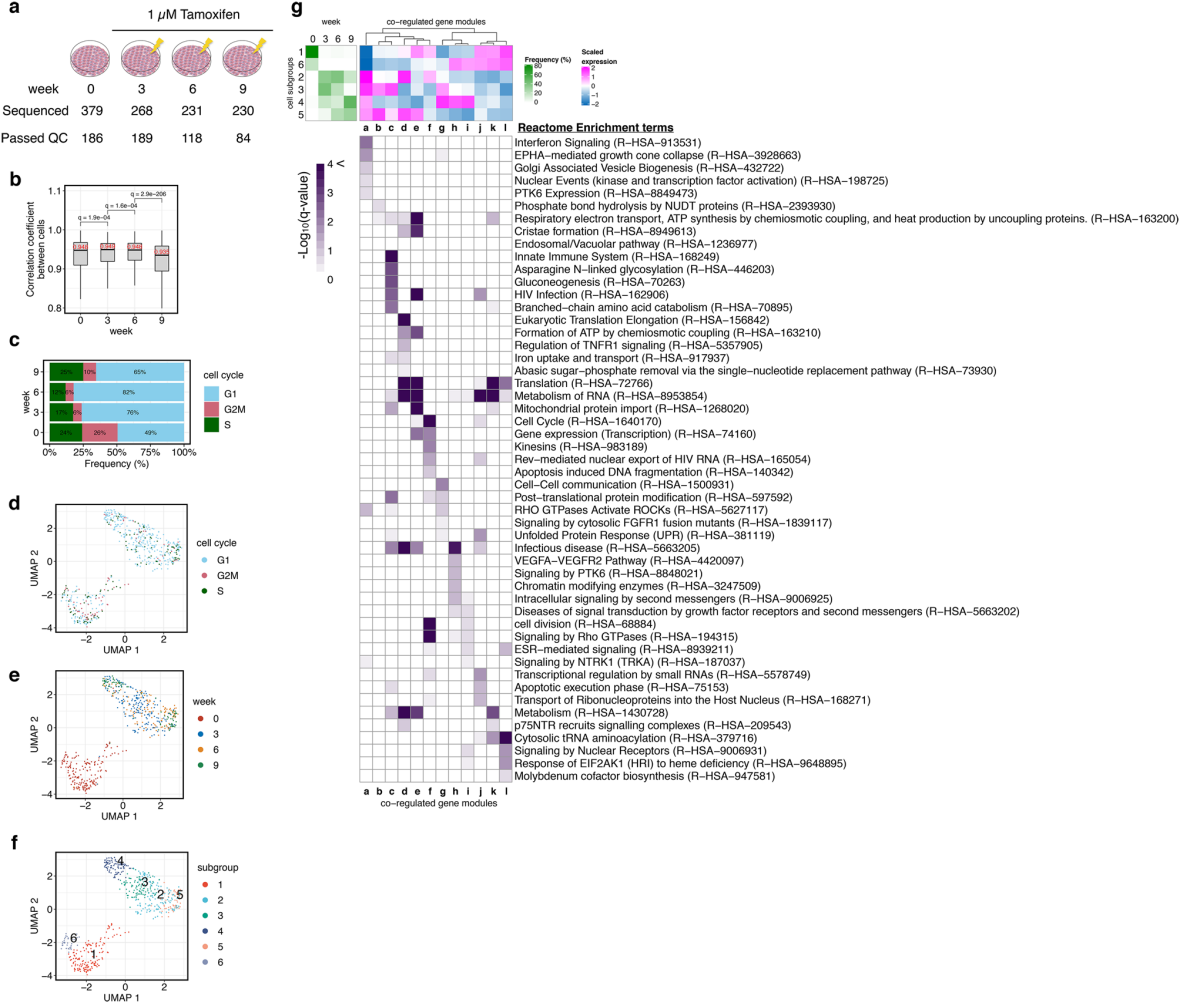
Single-cell RNA-seq analysis of TAM-resistant MCF-7 cells. (**a**) Schematic overview of the experimental procedure of single-cell RNA-seq. (**b**) Boxplot showing the distribution of the correlation coefficients of single-cell gene counting among cells at each time point. The median values are presented in red. The q-values were calculated using Wilcoxon rank-sum test. (**c**) Percentage of cells at different cell cycle stages at each time point. (**d**–**f**) Visualization of single-cell transcriptome data by UMAP. Single-cell data space was reduced to three dimensions, and the distribution of data was visualized using the first two dimensions. Cells were colored by estimated cell cycle stage (**d**), week (**e**), and subgroups (**f**). (**g**) Complex heatmap of cell subpopulation, co-regulated gene modules, and enriched functions. Top left heatmap presents the frequency of subgroups at each time point shown in (**f**). Top right heatmap presents the relative expression level of each gene module in each cluster. Bottom heatmap presents the enrichment terms in each gene module.

Because cell-to-cell heterogeneity of phenotypic features is a key mechanism of drug resistance^20^, we investigated TAM-induced changes in gene expression profiles at a single-cell level. On the basis of the results of the cell growth assay and bulk RNA-seq data, we focused on four time points: W0 (just before starting TAM treatment), week 3 (W3, at the beginning of the complete cell growth inhibition period), W6 (at the end of the complete cell growth inhibition period), and W9 (at the acquisition of TAM resistance) (Fig. 2a). RNA-seq analysis of 1,108 single cells yielded 577 high-quality single-cell gene expressions (Fig. 2a and Methods section). Averaged single-cell expression profiles were correlated with bulk expression despite the zero-inflated distribution (Supplementary Fig. S4). The Pearson correlation coefficient of 11,413 gene expression values between individual cells increased at W3, then gradually decreased at W6 and W9 (Fig. 2b). This changing pattern of correlation coefficient might suggest the selection of cells that can survive the TAM treatment and subsequently transition into multiple stable states.

To visualize cell-to-cell diversity in detail, we conducted uniform manifold approximation and projection (UMAP), one of the standard methods of dimensional reduction. Before drawing the UMAP plot, we calculated the probability score of cell cycle progression in each cell using Seurat 3 software^21^ to correct for the bias caused by the difference in the cell cycle stage (Fig. 2c). All cells were mapped on a three-dimensional UMAP plot and projected in two dimensions (Fig. 2d–f, and Supplementary Fig. S5). Single-cell data were roughly divided into two groups: W0 and the others (W3, W6, and W9). Cells were widely distributed in space at W3 and W6 but were localized in two separate regions at W9. These cells could be clustered into six subpopulations in the UMAP plot (Fig. 2f). Cells in subgroups 1 and 6 belonged to the W0 group, and these cells were strongly diminished by W3. By contrast, cells in subgroups 2 and 3 newly emerged at W3 and prevailed in those clusters until W6. Finally, at W9, these cells were split into two groups: one containing subgroup 4, and the other containing subgroup 5.

### Subgroup-specific gene modules and their functions

We first investigated marker genes in each subgroup. The top five genes showing the highest specificity scores were selected in each subgroup (Supplementary Fig. S6a). Subgroups 1 and 6 were the major groups at W0, and marker genes in these subgroups included typical ER pathway target genes such as *AREG*^22^ and *GREB1*^23^. This result showed that the transcriptional activity of ER for typical target genes was down-regulated in the other subgroups. Comparing with the marker genes in subgroups 1 and 6, the expression level of marker genes in other subgroups, especially that in subgroup 2, does not clearly distinguish the cells into the subgroups. Interestingly, almost all marker genes of subgroups 4 and 5 also showed high expression in subgroup 3, suggesting that the pre-resistant subgroup 3 could potentially mature into distinct resistant subgroups by rewiring the genetic network.

Next, we analyzed the genetic modules specifically expressed in each subgroup or each week (Fig. 2g, Supplementary Fig. S6b, and Supplementary Table S2). Subgroups 1 and 6 contained highly expressed gene modules j, k, and l, which are enriched in ESR-mediated signaling, unfolded protein response, and amino acid and nucleotide metabolism. On the other hand, the gene expression of module a was particularly low for these subgroups. Subgroups 2 and 3 were the major subpopulations in W3 and W6. Both these subgroups showed high expression levels of genes in module a, some of which are involved in interferon signaling, TGFβ signaling, and tight junctions. These enriched terms showed a strong resemblance to the early responsive cluster C in the bulk RNA-seq experiment (Fig. 1g,h). Subgroup 4, whose population was increased at W9, showed high expression levels of genes in modules g, h, and i, as shown in the heatmap. These genes encoded cell adhesion-related molecules such as integrin β4 (*ITGB4*), laminin β2 (*LAMB2*), and zyxin (*ZYX*), and some genes were involved in ROCK activation mechanisms. These modules also include several terms related to signal transduction, such as the VEGF pathway and thyroid hormone signaling. In addition, some chromatin remodeling enzymes and lysine-specific histone demethylases were included in module h. These results indicate that TAM-resistant cells in subgroup 4 showed higher activities of cell adhesion and migration, with an altered signaling pathway and epigenetic status. Subgroup 5, which represents another major population during W9, contained highly expressed genes in modules b, c, d, and e compared to that of subgroup 4. This result indicates that genes related to innate immune responses, oxidative phosphorylation, and translation are highly expressed in the cells in subgroup 5. In addition, module c contained genes related to carbon metabolism, especially the glycolysis/glycogenesis pathway, suggesting that cells in subgroup 5 exhibit unique metabolic adaptation to TAM-induced stress. Based on the aforementioned results, we found that TAM-resistant ER-positive breast cancer cells obtained from the same parental cell line could be divided into two types, one of which acquired the re-wired metabolic network (subgroup 5) and another acquired high expression levels of adhesion molecules with changing epigenetic status (subgroup 4).

### Trajectory analysis of TAM resistance

**Figure 3:**
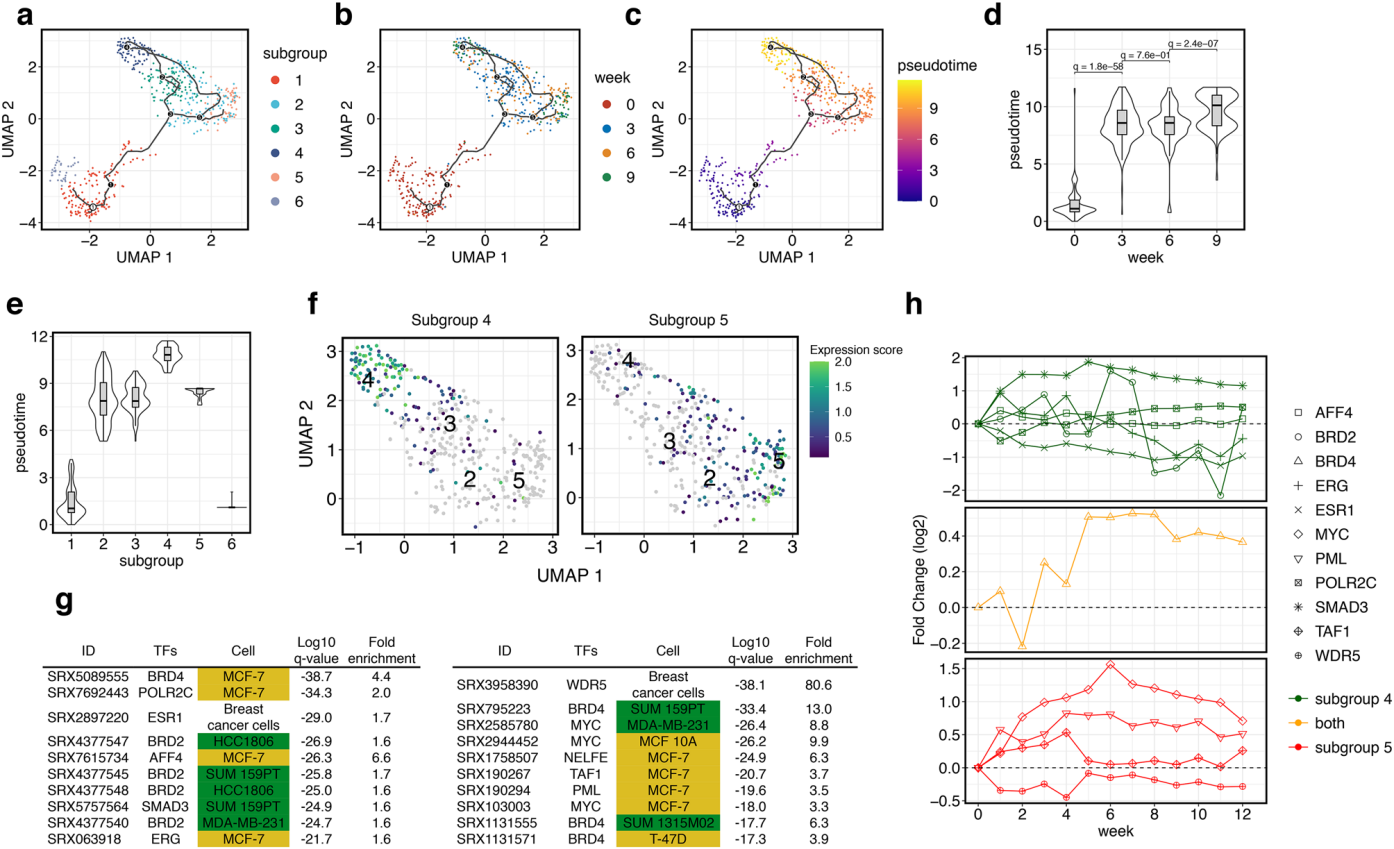
Trajectory analysis of the TAM resistance acquisition process. (**a**–**c**) Single-cell trajectory during the continuous TAM treatment. Graphs were colored by clusters (**a**), weeks (**b**), and pseudotime (**c**). Numbered circles in white and black indicate a root node and estimated branch nodes in the trajectory, respectively. (**d**,**e**) Violin plots showing the distribution of pseudotime in each week (**d**) and subgroup (**e**). The q-values in (**d**) are calculated using Wilcoxon rank-sum test. (**f**) Expression score of up-regulated genes in subgroup 4 (left) and subgroup 5 (right), considering the cell trajectory. (**g**) Upstream factor analysis of up-regulated genes displayed in (**f**). Top 10 q-value data are presented. Color represents subtypes of breast cancers: yellow, MCF-7 cells; green, triple-negative breast cancer (TNBC). (**h**) Time-series bulk gene expression patterns listed in (**g**). NELFE was not detected in bulk RNA-seq.

To confirm the cell transition trajectory into two different types of resistant subgroups, we conducted pseudotime analysis (Fig. 3a–c). The pseudotime of each cell calculated from the gene expression data was correlated with the sampling time after starting the continuous TAM treatment (Fig. 3d). The pseudotime of cells in subgroup 4 was higher than that of cells in subgroup 5, suggesting that cells in subgroup 4, showing high expression of epigenetic modulators, are more divergent from parental cells than cells in subgroup 5 (Fig. 3e). To indetify important molecules involved in the emergence of subgroups 4 and 5, we analyzed DEGs along the estimated cell trajectory. A total of 273 and 79 genes were detected as highly expressed genes in subgroups 4 and 5, respectively (Fig. 3f, Supplementary Tables S3 and S4). Then, we investigated the transcriptional regulators of the highly expressed genes in subgroup 4 and 5 using the ChIP-Atlas database^24^, which covers approximately all public ChIP-seq data (Fig. 3g), and verified the expression patterns of genes based on our analysis (Fig. 3h and Supplementary Fig. S7). The detected genes and expression patterns in bulk (Fig. 3h) and single-cell RNA-seq (Supplementary Fig. S7) were not completely correlated. These differences may be attributed to technical reasons, including the difference in sequencing protocols, depth per sample, and the lack of non-treatment control conditions in each week for single-cell data.

Prediction of proteins that bind near the transcriptional start site of DEGs in the trajectory to subgroup 4 showed that only 4 of the top 10 factors represented ChIP-seq data from MCF-7 cells, and most of the others were obtained from the triple-negative breast cancer (TNBC) cell line (Fig. 3g, left, shown in green). These results also suggest that most up-regulated genes in subgroup 4 are controlled by bromodomain-containing proteins, BRD4 and BRD2, which recognize acetylated histones and act as super enhancers^25,26^. In addition, our results also suggest the possible involvement of oncogenic transcription factors SMAD3 and ERG in the trajectory to subgroup 4. RNA expression levels of SMAD3 and ERG were up-regulated before W4 in bulk RNA-seq data (Fig. 3h, top and middle) and related molecular terminology (“Signaling by TGF-beta receptor complex” and “MAPK singaling pathway”) were detected in cluster C and E in bulk RNA-seq data, respectively (Fig. 1g,h). For single-cell data, the expression levels of BRD2, BRD4, and SMAD3 increased after TAM treatment (Supplementary Fig. S7). These results indicate that cells in subgroup 4 have different statuses due to epigenetic alteration, which is clearly distinct from that of parent MCF-7 cells; this result was consistent with the enrichment analysis of specific gene modules (Fig. 2g).

In subgroup 5, 7 of the top 10 factors were obtained from ER-positive breast cancer or normal cells (Fig. 3g, right), suggesting that subgroup 5 retained the transcriptional network of parental MCF-7 cells. ChIP-seq data from anti-WDR5 antibody showed the best q-value and fold enrichment score. Although the expression profiles of *WDR5* were not consistent between bulk and single-cell RNA-seq data (Fig. 3h, bottom, Supplementary Fig. S7), various binding molecules of WDR5, such as methylated histone H3 lysine 4 or MYC^27^, might regulate genes related to subgroup 5. Moreover, MYC was detected as a candidate estimated from ChIP-Atlas database for the upstream regulating of increased genes in subgroup 5. Among other candidate genes, *BRD4, TAF1*, and *PML* were up-regulated after TAM treatment in both bulk and single-cell data. These data indicate that MYC, TAF1, and PML may contribute to one of the emerging TAM-resistant subpopulations. Taken together, our analysis revealed key molecular candidates that drive two different TAM-resistant subgroups.

### Mathematical modeling of the TAM resistance acquisition process

**Figure 4:**
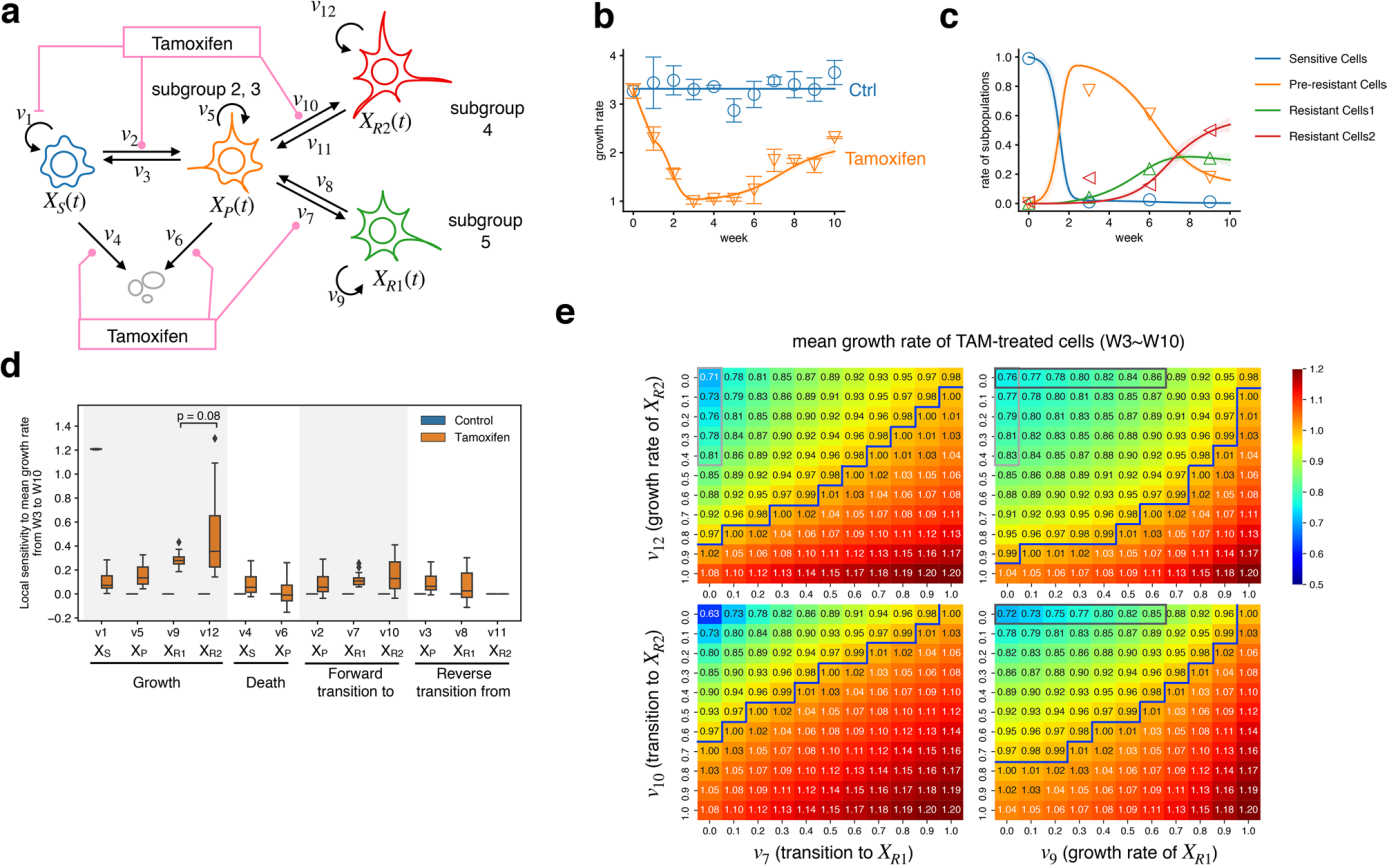
Ordinary differential equation-based model of the TAM resistance acquisition process. (**a**) Illustration of the model scheme. (**b**,**c**) Time-series analysis of changes in cell growth rate (**b**) and ratio of each subpopulation (**c**). Points: experimental data; error bars: standard deviation (SD) of experimental data; solid lines: averaged in silico simulation of 20 sets of parameters; shaded areas: SD of simulations. (**d**) Results of sensitivity analysis of the mean growth rate from week 3 (W3) to week 10 (W10) at each reaction; *v*_*1*_–*v*_*12*_. Error bars represent the SD of simulations with 20 set parameters. The p-value was calculated using Wilcoxon signed-rank test. (**e**) Heatmap of the mean growth rate of TAM-treated cells from W3 to W10, with different inhibitory intensity of growth rate of *X*_*R1*_ or *X*_*R2*_ and cell transition to *X*_*R1*_ or *X*_*R2*_. The X- and Y-axes indicate the remaining reaction rate (i.e., 1.0 means 0% inhibition, and 0.0 means 100% inhibition). Data represent the mean of simulations with 20 set parameters. Blue lines represent a border dividing mean growth rate of less than or equal to 1. Gray boxes indicate where conditions were compared in the main text and show q-values < 0.05.

We constructed a phenomenological mathematical model that reproduces the population-level dynamics of TAM resistance, based on cell trajectories obtained using pseudotime analysis, to estimate the relative contribution of TAM-mediated cell growth- and differentiation to the acquisition of resistance (Fig. 4a). This model comprises cell transformation among four major cell subpopulations as estimated by single-cell gene expression profiles: cells initially sensitive to TAM (*X*_*S*_, subgroups 1 and 6 in Fig. 3a), pre-resistant cells (*X*_*P*_, subgroups 2 and 3), resistant cells showing altered expression of metabolism-related genes (*X*_*R1*_, subgroup 5), and resistant cells with highly adhesive phenotype with changing epigenetics (*X*_*R2*_, subgroup 4). The state transitions between the four subpopulations were assumed to follow the graph structure estimated by pseudotime analysis (Fig. 3c): *X*_*S*_ cells change to *X*_*P*_ in the presence of TAM, and cells in *X*_*P*_ change to the two resistant states *X*_*R1*_ and *X*_*R2*_. The model also enables cell transitions in the reverse direction. In addition, we assumed that continuous TAM treatment accelerates the rate of forward cell transition rate in response to a cumulative history of TAM treatment and describe this acceleration as a sigmoid function of the integral of TAM. This assumption is based on previous findings that cell state transitions require the accumulation of genetic or epigenetic changes, which from the tipping point of resistance^15^. We explicitly considered extrinsic noise-mediated cell-to-cell variability by fitting 20 independent model parameter sets to two experimental time-course datasets describing cell proliferation and differentiation dynamics using the BioMASS computational framework^28^. Specifically, our model reproduced the experimentally observed dynamic distributions of both total cell growth rate in the presence of TAM (Fig. 1b) and the four different cell subpopulation proportions (Fig. 2g, heatmap in green) simultaneously (Fig. 4b,c, and Supplementary Fig. S8a).

We found two remarkable features of the well-fitting parameter distributions. First, the growth rate of subpopulation *X*_*R2*_ (rate constant of reaction *v*_*12*_) was significantly greater than that of *X*_*R1*_ (*v*_*9*_) (Supplementary Fig. S8b). This finding is consistent with the result that the subpopulation of *X*_*R2*_ expressed some cell division-related genes (Fig. 2g). Second, the parameter determining the steepness of the rection rate mediating the acquisition of TAM resistance was greater for *v*_*10*_ than that for at in *v*_*7*_ (Supplementary Fig. S8c). This implies that the cell transition from *X*_*P*_ to *X*_*R2*_ is more sensitive to cumulative TAM exposure time, which may be caused by the accumulation of epigenetic alterations. The finding is substantiated by the results of single-cell RNA-seq analysis, which showed that the genetic feature of *X*_*R2*_ displayed high expression levels of chromatin-modifying enzymes (Fig. 2g), and pseudotime analysis, in which *X*_*R2*_ was the most differentiated subtype compared with other subtypes (Fig. 3e). These results indicate that parameter inference using our phenomenological model, which is based on cell population dynamics, can help to successfully pinpoint underlying mechanisms (differential subpopulation-specific rates) which are consistent with empirical observations.

Using the fitted parameter sets (Supplementary Table S5) as nominal values, we then performed local sensitivity analysis to examine how much a given change in each single reaction affects the mean-over-time growth rate after the 3rd week (Fig. 4d). The results indicated that *v*_*12*_, the growth rate of *X*_*R2*_, was the most critical factor affecting the mean-over-time growth rate. On the contrary, neither the reverse transition from resistant cell types to *X*_*P*_ (or from *X*_*P*_ to *X*_*S*_) nor cell death caused by TAM was found to significantly affect the growth rate. Finally, we examined the effect of combination inhibition on two key biological processes, cell growth and forward state transition to two different subtypes, on TAM-resistant cell growth (Fig. 4e). The simulation results show that the mean-over-time growth rate was lower when the two parameters related to both subgroups were inhibited than when only the parameter related to one subgroup was inhibited. Additionally, combined inhibition of cell growth rate of both resistant subtypes inhibited growth rate < 1 at broader inhibitory ranges than the other intervention pairs (Fig. 4e, blue line). However, under the conditions in which the growth of one resistant subtype is repressed, complete inhibition of transition to another resistant subtype showed stronger regression than complete cell growth inhibition of the same subtype with statistical significance (Fig. 4e, comparison of the gray box). We cannot determine whether the difference is biologically significant, but the result may indicate that inhibition of cell state transition by, for example, epigenetic inhibitors, has the potential to be more effective than targeting the growth of the resistant subpopulation alone.

### Inhibition of molecules mediating the generation of two resistant subpopulations induces regression in the pre-resistant stage

**Figure 5:**
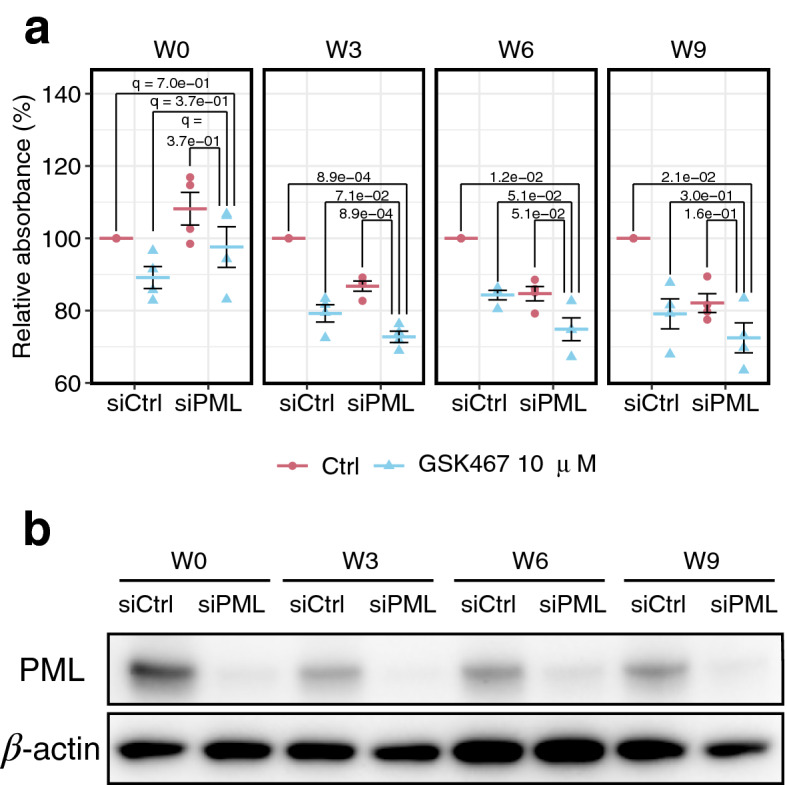
Experimental validation. (**a**) Effect of PML knockdown and KDM5 inhibitor GSK467 on the relative cell number of TAM-treated cells measured using MTT assay at each time point. siCtrl and siRNA indicate conditions in non-targeting siRNA-treated and those in siRNA targeting *PML*-treated, respectively. The data represent mean ± SE (n = 4). q-values were calculated using two-tailed Welch's test. (**b**) Protein expression levels of PML in each condition. β-actin presented as a loading control. Whole membrane images are presented in Supplementary Fig. S9.

Finally, we experimentally confirmed the hypothesis derived from pseudotime analysis and mathematical modeling that simultaneous intervention for the proliferation or transitions in subgroup 4, which undergoes epigenetic alterations via chromatin modification, and subgroup 5, wherein PML acts as an upstream regulator by effectively inhibiting the growth of the resistant cell population. Histone demethylase KDM5B is a candidate epigenetic modulator for subgroup 4, which reportedly modulates resistance to endocrine therapies by increasing transcriptional heterogeneity^7^. The combination of KDM5 inhibition and PML knockdown suppressed the cell growth of TAM-treated cells (W3, W6, and W9) but not that of cells not treated with TAM (W0) (Fig. 5). Particularly in W3 and W6, the combination inhibition was prone to suppress the cell growth more than PML knockdown or KDM5 inhibitor treatment alone. In addition, the growth inhibition in W3, the timing when the cell growth was completely inhibited by TAM (Fig. 1b), indicates that combined inhibition induces a decrease in cell numbers. Together, these results demonstrate that the inhibition of molecules important for resistant cell subgroups could induce regression before complete acquisition of TAM resistance.

## Discussion

In this study, we analyzed transcriptional changes in MCF-7 cells during continuous TAM treatment using both bulk and single-cell RNA-seq. The results of bulk RNA-seq analysis revealed several time-course patterns of gene expression during the continuous TAM treatment. A subset of genes, including clusters B and E, showed low or high expression immediately before acquiring the growing ability in the presence of TAM, respectively. It is reasonable to speculate that the recovery of gene expression levels in cluster B is accompanied by the recovery of growth rate because these genes included positive cell cycle regulators. The expression levels of these genes may be regulated by E2F families, suggesting that the growth of TAM-resistant cells also depends on the CDK4–E2F cell cycle machinery, supporting the effect of the CDK4/6 inhibitor on ER cells^29^. Combined with the expression pattern of ESR1, our results implied that the expression levels of E2F gene families are maintained by estrogen–ESR1-dependent signaling in the absence of TAM; however, this was superseded by other signaling pathways, such as central carbon metabolism-related HIF1 machinery, in TAM-resistant cells (Fig. 1g).

On the other hand, the significance in cluster E is rather difficult to interpret. Some groups have previously reported that interferon regulatory factor-1 (IRF1) is critical for TAM-mediated apoptosis, and its related pathway is also up-regulated in TAM-treated cells^30^. IRF1 was shown to induce apoptosis in breast cancer cells^31^. However, another group showed that interferon-responsive genes are up-regulated in both TAM-resistant and radioresistant MCF-7 cells and contribute to cross resistance^32^. These previous reports imply that the bilateral function of interferon signaling may accelerate the adaptation of cancer cells to the TAM-treated condition by increasing cell-to-cell variability (Fig. 2b). Non-genetic cell-to-cell variability, believed as a major contributor to the production of outlier cells and can adapt to severe conditions^33,34^, could play an important role in the acquisition of TAM resistance under our experimental conditions because few genes were mutated at the time when genes in cluster E were up-regulated^15^ (Fig. 1f). Previous studies show contradictory results on the relationship between chemosensitivity and FoxO-autophagy signaling. It has been reported that 4-hydroxytamoxifen induces autophagic cell death^35,36^; however, another group reported that inhibition of autophagy restored the responsiveness to anti-estrogen therapy. In our single-cell RNA-seq analysis, cells in subgroup 4 showed high expression levels of autophagy-related genes (Supplementary Fig. S6b). Our results suggest the possibility that the modulation of autophagy and interferon signaling in early phases of endocrine therapy prevents the transition of cells to resistant types.

Several studies showed that TAM is localized to mitochondria and endoplasmic reticulum and shows non-genomic toxicity by inhibiting the electron transport chain complexes^37,38^. Some results in our transcriptomic analysis can be explained by such estrogen-independent mode of action of TAM. The overrepresentation of genes related to translation in cluster C and that of genes involved in the detoxification of reactive oxygen species, which are mainly produced in mitochondria, in cluster D (Fig. 1g,h) can be interpreted as a protective response for the dysfunction of these organelles. High expression levels of ribosomal and mitochondrial genes (modules d and e) were also detected in TAM-resistant subgroup 5 by single-cell RNA-seq analysis (Fig. 2g). Another phenomenon related to mitochondrial dysfunction was the up-regulation of glycolytic pathway enzymes induced by the HIF1 signaling pathway (Fig. 1h), which was coincident with the growth ability of cells in the presence of TAM (Fig. 1b). We detected the overexpression of genes encoding glycolytic and gluconeogenetic enzymes in subgroups 3 and 5. Indeed, MYC, which drives a gene expression of ribosomal proteins^39^, hexokinase 2, and lactate dehydrogenase^40^ was increased during the time-course, and was predicted as one of the main regulators of resistant subgroup 5 (Fig. 3g). These results suggest that subgroup 5 genes overcome the non-genomic toxicity of TAM by up-regulating the ribosomal and mitochondrial functions via HIF1 or MYC activity.

Our single-cell RNA-seq analysis suggested the existence of two different resistant subpopulations and the role of important molecules in the emergence of each resistant subpopulation. Subgroup 5 was predicted to be initiated by the activity of TAF1 and PML molecules, in addition to MYC (Fig. 3g). Interestingly, the second bromodomain-specific inhibitor of TAF1 represses *MYC* expression, and its effect is synergistic to the BRD4 inhibitor^41^. On the basis of the results of this and previous studies, we infer that the differentiation of pre-resistant cells to resistant subgroup 5 requires TAF1 and BRD4 activity for up-regulating *MYC* gene expression. PML is believed to possess tumor-suppressing activity by controlling the cell cycle and apoptosis^42^; however, recent studies revealed that PML is overexpressed and promotes metastasis, especially in TNBC^43,44^. This bimodal character of PML was also detected in our experiments; PML knockdown decreased cell growth in TAM-treated cells but increased it in cells without TAM treatment (Fig. 5). Although the overexpression of PML in luminal types is uncommon, silencing PML functions elicits not only growth suppression in TNBC^45^ but also oncosphere formation, a readout of self-renewal potential, in PML-overexpressing luminal type breast cancers^46^. In addition, PML overexpression in MCF-10A cells promotes fatty acid oxidation and ATP production via the tricarboxylic acid cycle^44^. Taken together, these results suggest that TAM-resistant cells in subgroup 5 are similar to proliferative cancer stem cells, which exhibit self-renewal potential and rely on both oxidative phosphorylation and glycolytic metabolism^47^. The second resistant subgroup (subgroup 4) showed high expression levels of prostate cancer-related genes and chromatin-modifying enzymes (Fig. 2g and Supplementary Fig. S6b, module h). Some of the TAM-resistant specimens showed the overexpression of androgen receptor (AR), and exogenously AR-overexpressed MCF-7 cells resistant to TAM-induced growth inhibition^48^. This study is consistent with our results that one resistant subpopulation acquired new TAM-resistant features by AR signaling and histone-modifying enzymes.

In summary, our time-series single-cell sampling and multidimensional data analysis highlighted that the acquisition of drug resistance relies on heterogeneity and emphasized the importance of multiple molecules in phenotype transitions. Our approach reproduces the characteristics of the emergence of TAM-resistant cell populations by proposing a mathematical model of subpopulation dynamics based on cell trajectories obtained by single-cell analysis. Further, analysis of our phenomenological model allowed us to pinpoint key mechanisms differentiating the two TAM-resistant subpopulations (differential proliferation rates) and to devise combinatorial intervention strategies that effectively halt the progression to resistant states. Moreover, we empirically confirmed these two model predictions in our bulk dynamic gene expression data set by the double inhibition of two molecular mediators of TAM resistance acquisition.

The combination of enrichment (Fig. 3g) and sensitivity analyses (Fig. 4d) would enable the prediction of target subpopulations and important molecules for tumor growth inhibition and aid in prioritizing the predicted molecular targets. However, there are some limitations to our research. First, the model does not completely reproduce the in vivo environment of breast cancer because all experimental data in this study were obtained from two-dimensional cell culture experiments. In a three-dimensional in vivo environment, cell growth is spatially restricted and cell-to-cell communication within the microenvironment must also be considered. To understand the mechanism of TAM resistance in vivo, it will be necessary to combine our approach with modeling studies of spatially constrained three-dimensional cancer cell growth in breast ducts^49^ and maintenance of cancer cells in their niche^50^. Second, the generalizability of our findings is unclear because the single-cell sequencing data correspond to a single experimental condition using MCF-7 cells and TAM simultaneously. Validation experiments with KDM5 inhibitor and PML knockdown (Fig. 5) suggest that the two resistant states we observed are reproducible, but our results do not preclude the existence of other resistant subpopulations. In addition, data analysis using multiple cell line experiments or patient-derived samples is necessary to discuss the similarities and diversity of resistance acquisition processes in breast cancer. Accordingly, our approach combining scRNA-seq with mathematical modeling should be extended to address other data derived from different backgrounds, such as different concentrations of estrogenic compounds to account for pre/menopausal status^51^ and various breast cancer cells to account for the diversity of genetic mutations. Future studies also should address drug-independent fluctuation of subpopulations. Despite these issues, the combination of single-cell RNA-seq analysis of cancer cells with mathematical modeling could contribute to the understanding of the process of drug resistance acquisition and to designing novel treatment strategies.

## Methods

### Cell culture

Human breast adenocarcinoma MCF-7 cells were cultured in Dulbecco's modified Eagle's medium supplemented with 10% fetal bovine serum and antibiotics, as described previously^15^.

### Cell growth assay

Approximately 1 × 10^6^ MCF-7 cells were seeded in a 100-mm dish containing 10 mL of culture medium supplemented with or without 1 µM TAM. After a week, cells were detached and collected with trypsinization, and the concentration of the cell suspension was measured using a hemocytometer. The cell growth rate per week was calculated by dividing 1 × 10^6^ with the total number of cells in each cell suspension.

### Cell cycle analysis by flow cytometry

MCF-7 cells were trypsinized, washed with phosphate-buffered saline (PBS), and fixed with 80% ethanol. Subsequently, the fixed cells were washed with PBS and stained with PI staining solution (BD bioscience, CA, U.S.A.) for 15 min. The PI-stained cells were subjected to flow cytometry using the FACSCanto II Flow Cytometer (BD bioscience), and the number of cells at each cell cycle stage was analyzed using the FlowJo 7.6.5 software.

### Gene silencing with siRNA

Gene silencing in MCF-7 cells performed by reverse transfection of 30 nM of SMARTpool ON-TARGETplus siRNA (Horizon Discovery, UK), targeting PML (L-006547-00) and Non-targeting Pool (D-001810-10) in 96-well-plates, as described previously^52^.

### MTT assay

Cells were seeded at 3 × 10^4^ cells per 96-well-plate with reverse transfection of siRNA. After overnight incubation, cells were treated with compounds at indicated concentration. After 96 h, cells were treated with 0.5 mg/ml of MTT, and incubated for 4 h. Then, the medium was completely removed, and the insoluble formazan was resuspended with 100 µl of DMSO. Finally, absorbance at 570 nm (objective) and 650 nm (reference) was measured using a microplate reader.

### Western blotting

Cells were collected and lysed with RIPA buffer (50 mM Tris–HCl pH 7.4, 150 mM NaCl, 1% NP-40, 0.1% w/v SDS, and 0.5% w/v sodium deoxycolate) containing both protease inhibitors and a phosphatase inhibitor cocktail (Nacalai Tesuque, Japan). The lysates were centrifuged and supernatants were recovered. After determining the protein concentration in each lysate, and boiling in a quarter volume of loading buffer (125 mM Tris–HCl pH 6.8, 25% v/v glycerol, 5% SDS, 0.25% w/v bromophenol blue, and 5% v/v 2-mercaptoethanol), samples were then electrophoresed in a polyacrylamide gel. Proteins were transferred onto a PVDF membrane, and immunoblotted. Antibodies employed for immunoblotting were anti-PML antibody (ab72137, Abcam, UK) and (sc-47778, Santa Cruz Biotechnology, TX, U.S.A.).

### Bulk RNA-seq analysis of TAM-resistant cells

Bulk RNA-seq data of MCF-7 cells have been published previously^15^. Briefly, RNA was extracted from MCF-7 cells treated with or without 1 µM TAM using QIAshredder (QIAGEN, Netherlands) and RNeasy Mini Kit (QIAGEN) every week up to 12 weeks, and then used for RNA-seq analysis. Different sequencing methods were used, which resulted in either 100-bp paired-end reads or 36-bp single-end reads (Supplementary Fig. S1). To remove the influence of different sequencing methods, we used only the first 36 bp of the first single-end read of paired-end data. After removing adaptor sequences and checking sequence quality using Trim Galore (https://github.com/FelixKrueger/TrimGalore/tree/0.6.7), the reads were aligned to the human reference genome (version GRCh38), and the read number counted by featureCounts^53^ without multi-mapping and multi-overlapping. The expression level of each gene was quantified as transcripts per million (TPM). TPM data of each sample were used for PCA to analyze the variability and reproducibility of the data (Supplementary Fig. S1). Comparing gene expression profiles between TAM-treated and control condition at each time point, DESeq2^54^ were used for calculating fold change (FC). In order to reduce data dimensions, genes which did not show significant changes (determined by the following cutoff: q-value < 0.001 and |log2FC| > 0.5) at least three time point were filtered out for further analysis. As a consequence, 6982 genes were used for cluster analysis. Then, the log2FC values of all genes at W0 were set as a theoretical zero value. Hierarchical clustering of z-score of log2FC values (13 time points) in those genes was performed by using the method of Ward’s linkage based on the Pearson’s correlation distance (1 − Pearson’s correlation coefficient).

### Enrichment analyses

All enrichment analyses except Fig. 3g were carried out using the Targetmine platform^55^. Redundant enrichment terms, shared by > 70% of the genes of interest, were removed from the results, and the term with the lowest q-value was retained. The enrichment analysis of upstream transcriptional regulators (Fig. 3g) was performed using the ChIP-Atlas database (https://chip-atlas.org)^24^ under the following settings: antigen class, “TFs and others” and cell type class, “Breast.”

### Single-cell RNA-seq analysis of TAM-resistant cells

Single-cell RNA-seq in this study was performed in single replicate. Single cells were separated using the ICELL-8 system (Takara Bio, Shiga, Japan). MCF-7 cells treated with or without continuous TAM were trypsinized and collected following dilution with the culture medium. The cells were then washed twice with cold PBS and stained with Hechest33342 (5 µg/mL) and PI (1 µg/mL) for 15 min. After staining, the cells were diluted to a concentration of 20,000 cells/mL and loaded into the ICELL-8 single-cell system. Then, cDNA was prepared using 3′DE reagents (Takara Bio), according to the manufacturer's instructions, and subjected to 100-bp paired-end sequencing on the Illumina HiSeq 3000 platform (Illumina, CA, U.S.A.). Mapping of sequence reads to the human reference genome sequence and counting genes were carried out using the mappa and hanta software (Takara Bio). The gene count data of individual cells were cleaned using the Seurat 3 software^21^. A series of quality controls were implemented. First, any gene expressed in < 5 cells at < 5 counts per million was removed. Cells with < 1500 detected genes and > 25% mitochondrial genes were filtered out. After filtering, the count data matrix consisting of 11,413 genes and 186, 189, 118, and 84 cells at weeks 0, 3, 6, and 9, respectively, was obtained. Next, any bias due to differences in the cell cycle stage was removed using the function *CellCycleScoring* and cell cycle gene set, and the effect of cell cycle phases on gene expression data was regressed. The data were imported into the Monocle 3 software^56^, and the data dimensions were reduced to three with UMAP. Then, cells were categorized into multiple classes. Gene module and pseudotime analyses were carried out using the Monocle 3 software, according to the developer's instructions (https://cole-trapnell-lab.github.io/monocle3/). Marker genes in each subgroup were calculated with the “top_markers” function (Supplementary Fig. S6). The pseudotime of each cell was calculated on the basis of the relative distance from open circle #1 (set as pseudotime = 0) (Fig. 3c). DEGs along with an estimated cell trajectory were calculated by applying the “graph_test” and “find_gene_modules” functions to cell subsets of groups 2, 3, 4, and 5 (Fig. 3f).

### Mathematical simulation

The mathematical model comprised 12 ordinary differential equations with 19 parameters. In this model, cell growth was assumed to follow logarithmic growth, and cell state transitions were assumed to follow the trajectory predicted by pseudotime analysis (Fig. 4a). Details of the equations are summarized in Supplementary Table S5. Mathematical simulation and parameter search were performed using the BioMASS platform^28^. During the parameter search process, we attempted to minimize the weighted sum of squared percentage errors (*wSSPE*):

$$wSSPE= \sum_{i=1}^{n}{\left(\frac{{x}_{sim, i}-{x}_{exp, i}}{{x}_{exp, i}+0.1}\right)}^{2}$$

where *wSSPE* is an objective function; *n* is the number of obtained data points to be fitted such as growth rate and rate of subpopulation at each time point and treatment; *x*_*sim*,*i*_ and *x*_*exp*,*i*_ are the *i*th simulation and experimental data, respectively. Importantly, “weighted” SSPE (calculated by adding 0.1 to the denominator of objective function) was used instead of normal SSPE to achieve two purposes simultaneously: escaping division by zero and fitting the simulation results to two experimental datasets with different range limits. In this study, a total of 38 (*n* = 38) data points, with 11 from growth rate of TAM-treated cells (W0–W11), 11 from parental cells, and 16 from the ratio of four subgroups in four time points (W0, W3, W6, and W9) were analyzed. Note that growth rate data in parental cells do not constrain parameters working in TAM-treated conditions and the degree of freedom of the subpopulation rate at each time point is 3. Therefore, the parameters in the model are practically constrained by 23 data points.

### Sensitivity analysis

The single parameter sensitivity of each reaction is defined as follows:

$${s}_{i}\left(q\left({\varvec{v}}\right),{v}_{i}\right)= \frac{\partial \mathrm{ln}(q\left({\varvec{v}}\right))}{\partial \mathrm{ln}({v}_{i})}= \frac{\partial q\left({\varvec{v}}\right)}{\partial {v}_{i}}\cdot \frac{{v}_{i}}{q\left({\varvec{v}}\right)},$$

where *v*_*i*_ is the *i*th reaction; ***v*** is a reaction vector (***v*** = *v*_*1*_, *v*_*2*_, …); and *q*(***v***) is a target function. *q*(***v***) we considered in this study is the mean-over-time growth rate after W3 described below:

$$\frac{\sum_{t=4}^{10}{X}_{total}(t)/ {X}_{total}(t-1)}{7},$$

where *t* denotes the time (week), *X*_*total*_(*t*) means the whole cell number at the time *t*. The sensitivity of each reaction was calculated with 1% increase in the reaction rate using the BioMASS platform^28^.

### Statistics and reproducibility

The number of replicates were as follows: Bulk RNA-seq, 5; single-cell RNA-seq, 1; and cell growth and cell cycle experiment, 2 or more than 2 (depending on experiments and described in the corresponding figure legend). The statistical comparisons between two samples in cell-biological experiments observing the representative value of a cell population, i.e., growth assay, cell cycle assay, and MTT assay, were performed using independent two-tailed Welch’s test. Those from single-cell data not showing Gaussian distributions were comapred using Wilcoxon rank-sum test. The comparison between simulation parameters were performed using Wilcoxon signed-rank test. For multiple test correction, the Benjamini–Hochberg method was used and adjusted p-values were presented as q-values.

## Supplementary Information

Supplementary Figures.

Supplementary Table S1.

Supplementary Table S2.

Supplementary Table S3.

Supplementary Table S4.

Supplementary Table S5.

## Data Availability

The raw bulk RNA-seq and single-cell RNA-seq data are deposited in the DNA Data Bank of Japan (DDBJ) and available under the accession numbers DRA004349 and DRA009126, respectively.
