# Supplementary material for: A combination approach of pseudotime analysis and mathematical modeling for understanding drug-resistant mechanisms
Source: Sci Rep. 2021 Sep 16;11:18511. doi: 10.1038/s41598-021-97887-z (PMC8445918; doi:10.1038/s41598-021-97887-z)
Supplement: Supplementary file 1 — Supplementary Figures. [file 41598_2021_97887_MOESM1_ESM.pdf]

A combination approach of pseudotime analysis and mathematical modeling for understanding drug-resistant mechanisms

Shigeyuki Magi[1, 2, 3*], Sewon Ki[2], Masao Ukai[4], Elisa Domínguez Hüttinger[5], Atsuhiko T Naito[3], Yutaka Suzuki[6], Mariko Okada[1, 2, 4, 7, 8*]

1. Laboratory of Cell Systems, Institute for Protein Research, Osaka University, Osaka 565-0871, Japan
2. Laboratory for Integrated Cellular Systems, RIKEN Center for Integrative Medical Sciences (IMS), Yokohama 230-0045, Japan
3. Division of Cell Physiology, School of Medicine, Toho University, Tokyo 143-8540, Japan
4. Graduate School of Medical Life Science, Yokohama City University, Yokohama 230-0045, Japan
5. Departamento de Biología Molecular y Biotecnología, Instituto de Investigaciones Biomédicas, Universidad Nacional Autónoma de México, Ciudad Universitaria, 04510, México, México
6. Department of Computational Biology and Medical Sciences, Graduate School of Frontier Sciences, The University of Tokyo, Chiba 277-8562, Japan
7. Center for Drug Design and Research, National Institutes of Biomedical Innovation, Health and Nutrition, Ibaraki, Osaka, 567-0085, Japan
8. Institute for Chemical Research, Kyoto University, Uji, Kyoto, 611-0011, Japan

* Correspondence to: Shigeyuki Magi, E-mail: shigeyuki.magi@med.toho-u.ac.jp and Mariko Okada, E-mail: mokada@protein.osaka-u.ac.jp

Supplementary figure S1. Principal component analysis (PCA) of transcripts per million (TPM) of genes during the acquisition process of tamoxifen resistance.

Supplementary figure S2.
MA-plots showing the log2-fold change and mean expression level from DESeq2 analysis.

Supplementary figure S3.
Relationship between time-course changing in cell growth rate and gene expression.

Supplementary figure S4.
Scatter plots showing the correlation between bulk RNA expression and averages of single cell RNA level.

Supplementary figure S5.
3-D UMAP plot of single cell RNA-seq data.

Supplementary figure S6.
Marker genes (a) and KEGG pathway enrichment (b) of single cell analysis.

Supplementary figure S7.
Time series single cell expression patterns listed in Figure 3g.

Supplementary figure S8.
Features of mathematical model presenting tamoxifen resistance.

Supplementary figure S9.
Raw images in Figure 5b before cropping.

Supplementary table S1.
Clustering results of bulk RNA-seq data

Supplementary table S2.
Gene modules of single cell RNA-seq data

Supplementary table S3.
Highly expressed genes in subgroup 4

Supplementary table S4.
Highly expressed genes in subgroup 5

Supplementary table S5.
Initial conditions and equation of cell fate decisions in the model

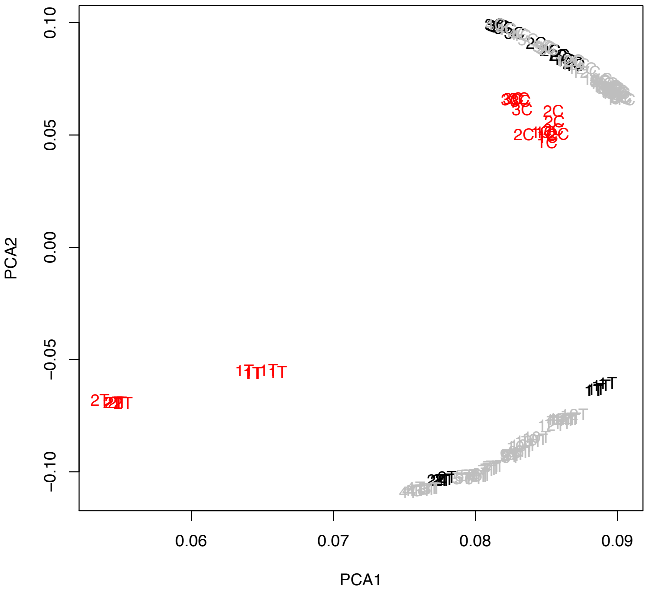

Supplementary figure S1. Principal component analysis (PCA) of transcripts per million (TPM) of genes during the acquisition process of tamoxifen resistance. Top graph shows PCA plot of all samples; each annotation means culture duration (week) and condition (C: control, T: 1 μM of tamoxifen-treated). Gray: data from 36 bp single read, Red; data from 100 bp paired end, Black: data from 100 bp paired end but only use 36 bp in the 1st fastq file to remove the influence of different sequencing method.

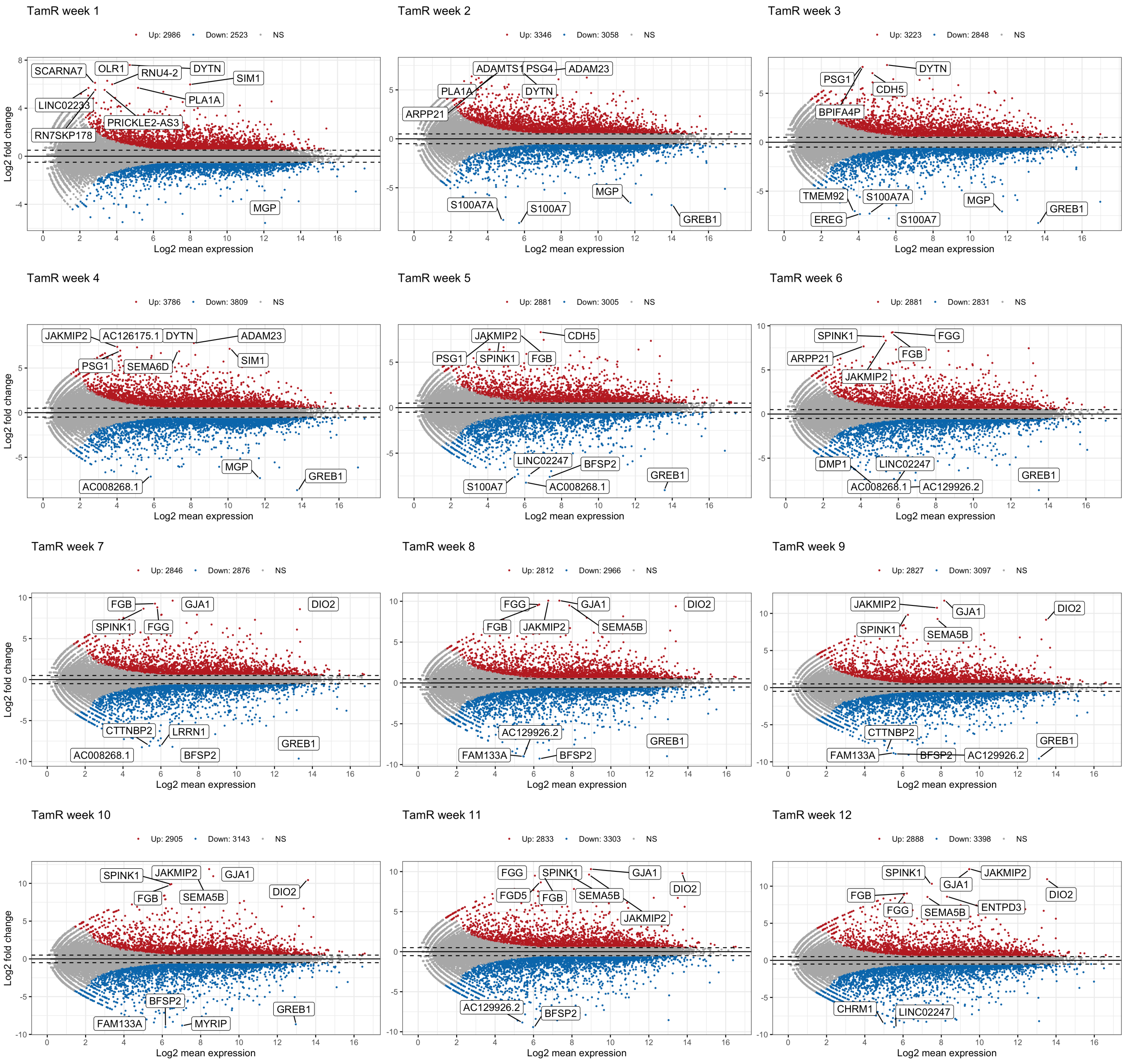

Week	1	2	3	4	5	6	7	8	9	10	11	12
DEGs (Up)	2986	3346	3223	3786	2881	2881	2846	2812	2827	2905	2833	2888
DEGs (Down)	2523	3058	2848	3809	3005	2831	2876	2966	2097	3143	3303	3398

Supplementary figure S2. MA-plots showing the log2-fold change and mean expression level from DESeq2 analysis. Colored dots represent significantly increased (red) or decreased (blue) genes (FDR < 0.001) and horizontal dashed lines represent the log2FC threshold of +/- 0.5. Genes showing Top 10 |log2FC| are annotated.

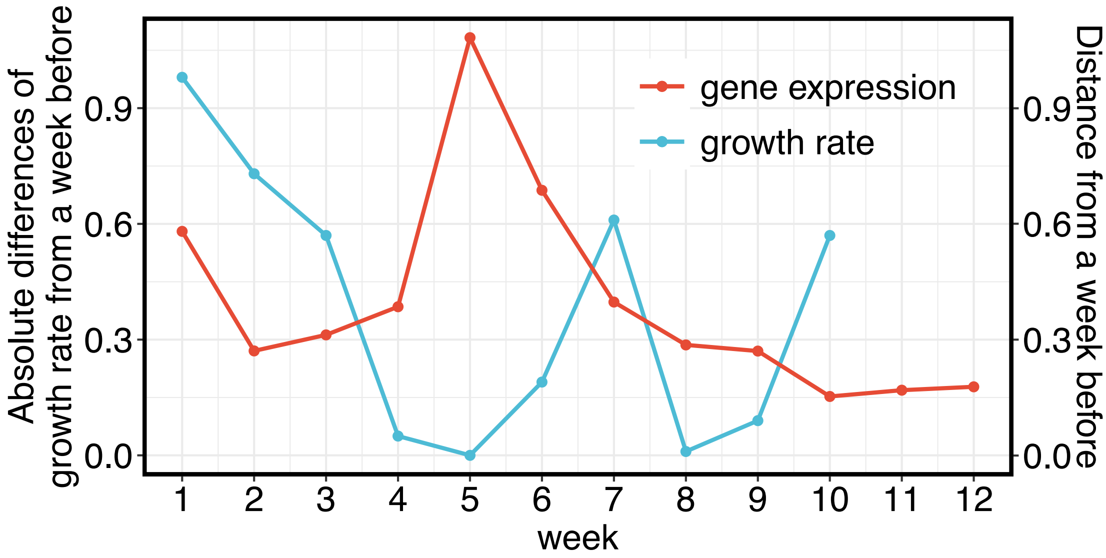

Supplementary figure S3. Relationship between time-course changing in cell growth rate and gene expression.

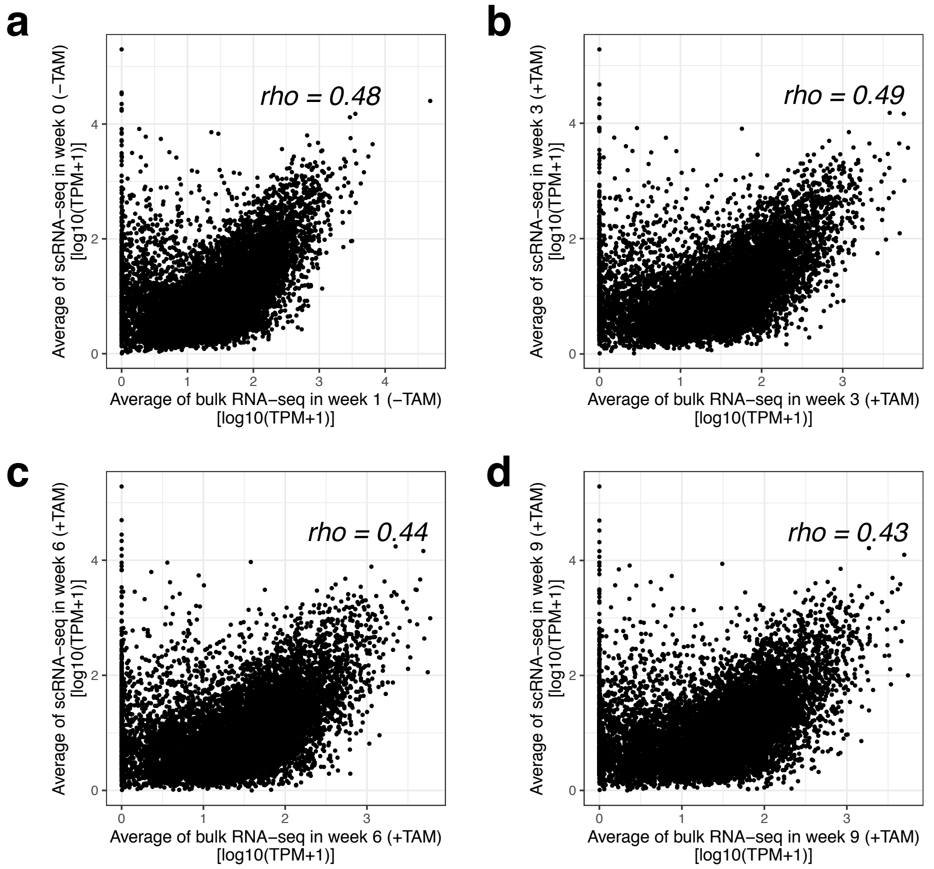

Supplementary figure S4. Scatter plots showing the correlation between bulk RNA expression and averages of single cell RNA level. (a) non-treated condition. **(b-d)** continuous treatment of tamoxifen for 3 **(b)**, 6 **(c)**, and 9 **(d)** weeks. rho means Spearman's rank correlation.

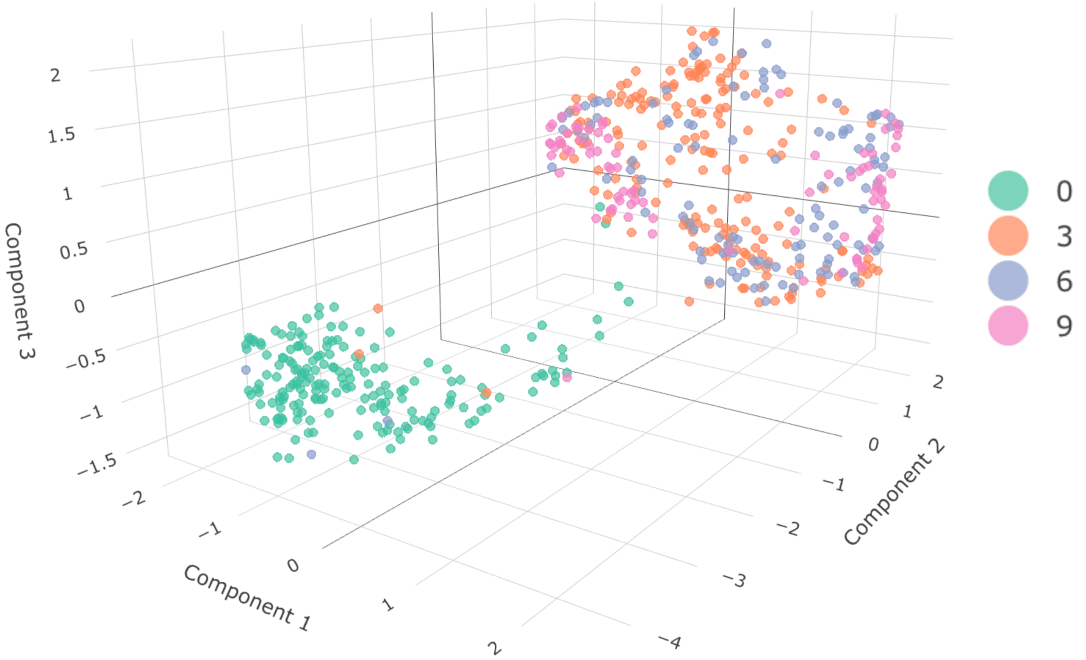

Supplementary figure S5. 3-D UMAP plot of single cell RNA-seq data. each point indicates single cell collected at 0, 3, 6, or 9 week after tamoxifen treatment.

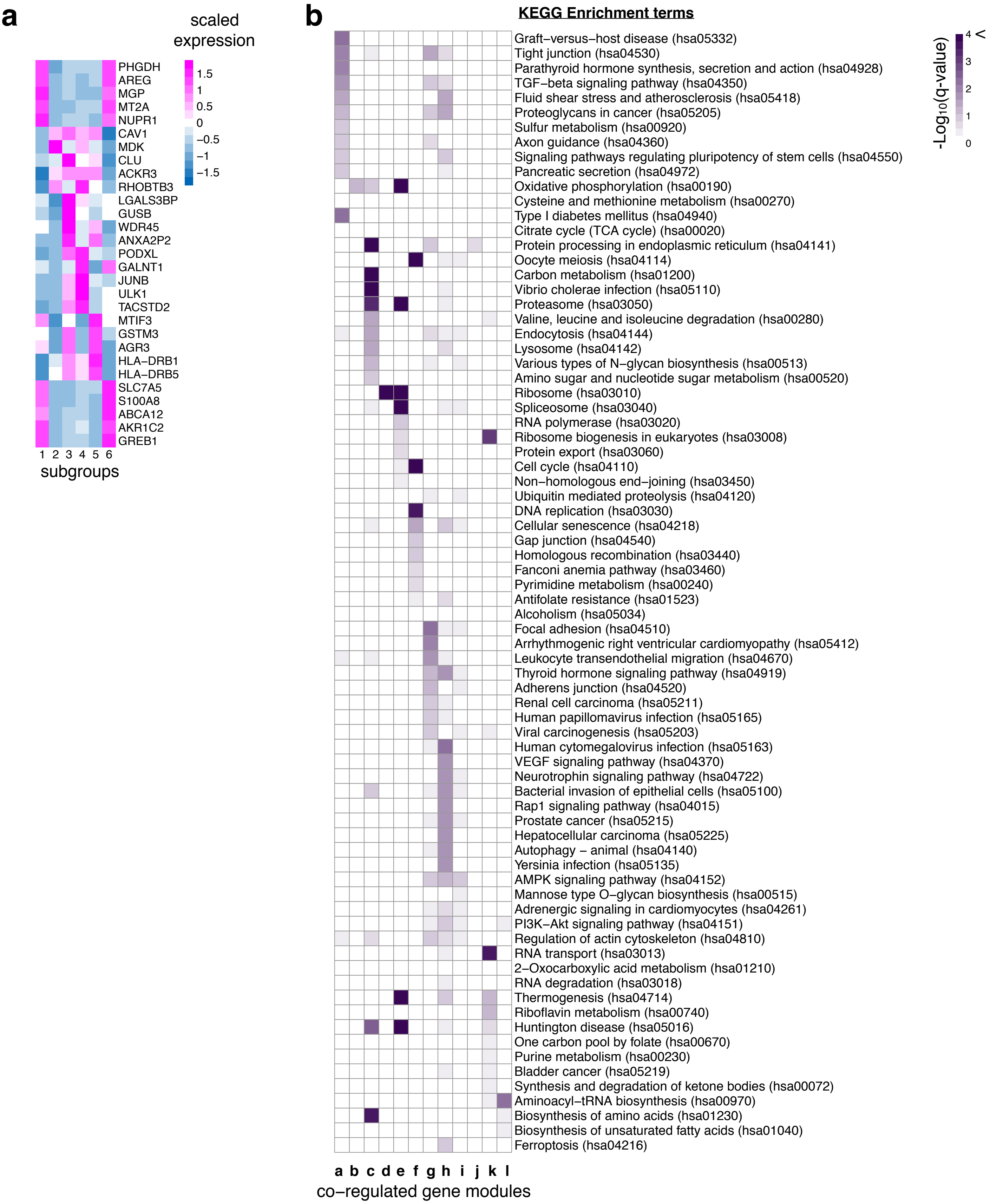

Supplementary figure S6. Marker genes (a) and KEGG pathway enrichment (b) of single cell analysis.

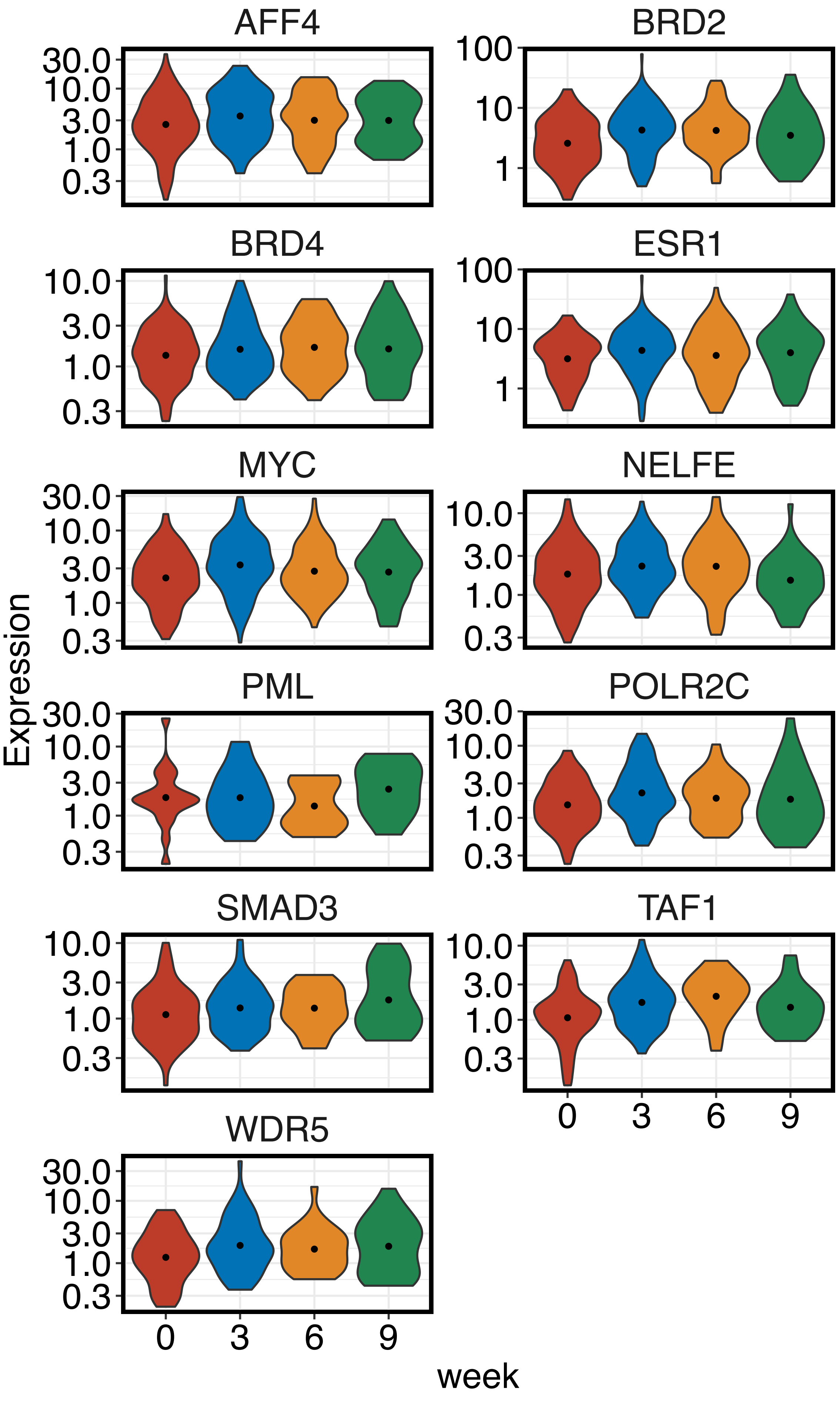

Supplementary figure S7. Time series single cell expression patterns listed in Figure 3g.
ERG was not detected in single cell RNA-seq.

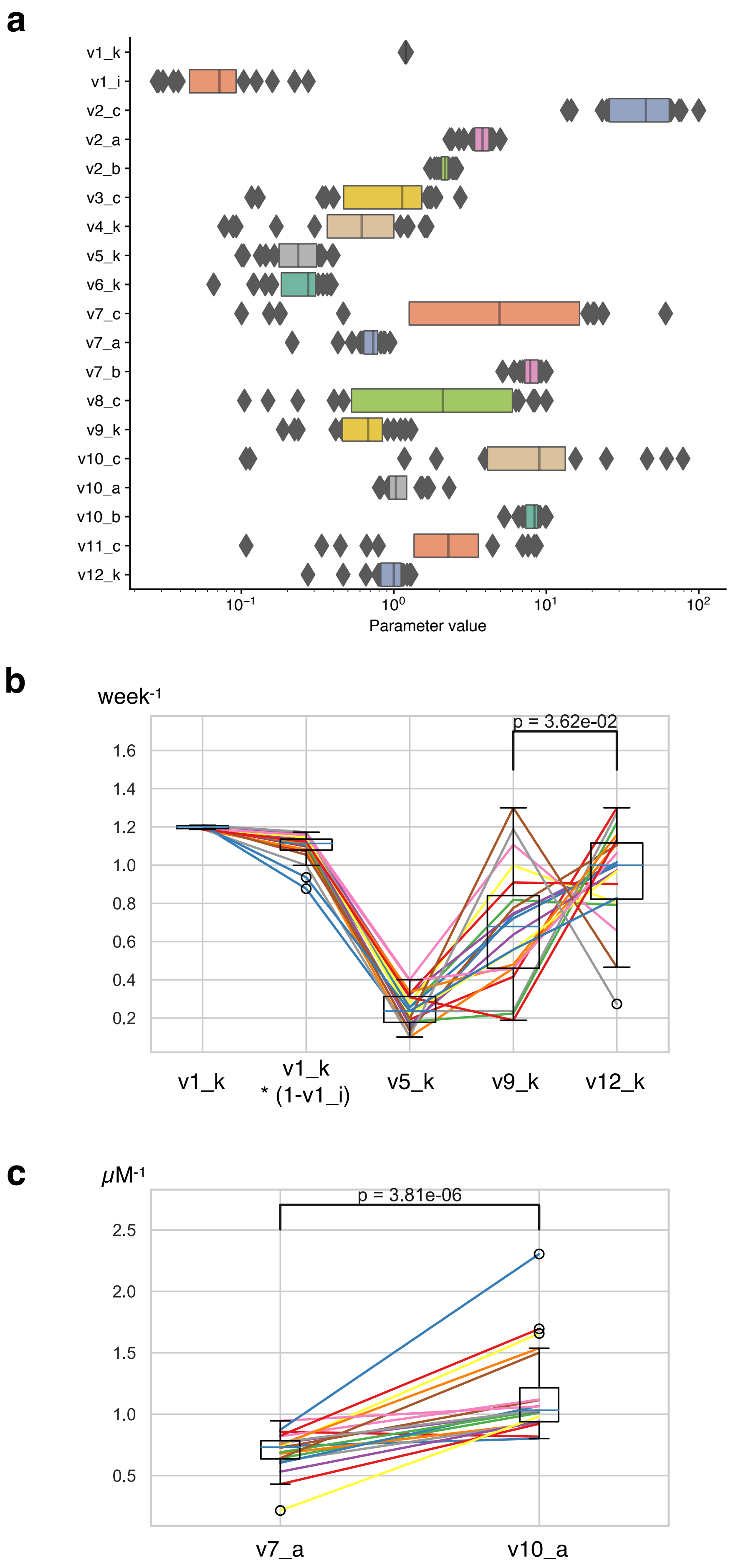

Supplementary figure S8. Features of mathematical model presenting tamoxifen resistance. (**a**) Ranges of obtained 20 parameter values. (**b**) Comparison of growth constants of each subpopulation. (**c**) Comparison For (**b**) and (**c**), each colored line means parameter combinations at each parameter set. p-values are calculated from Wilcoxon signed-rank test.

a

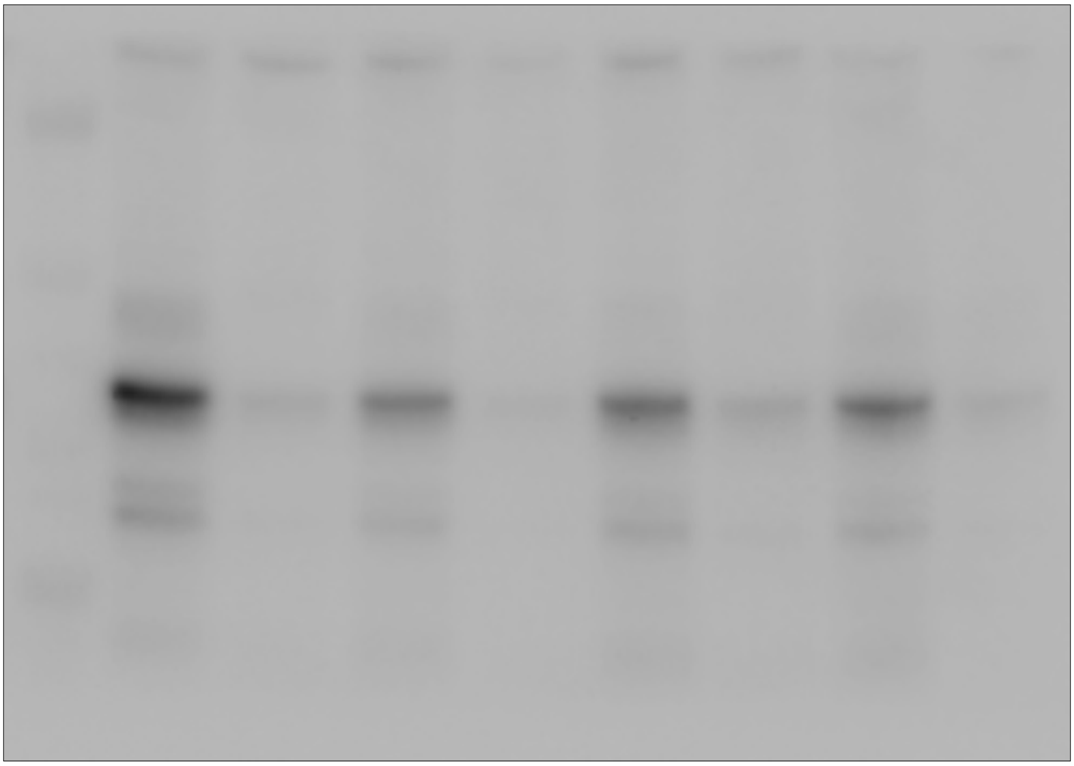

b

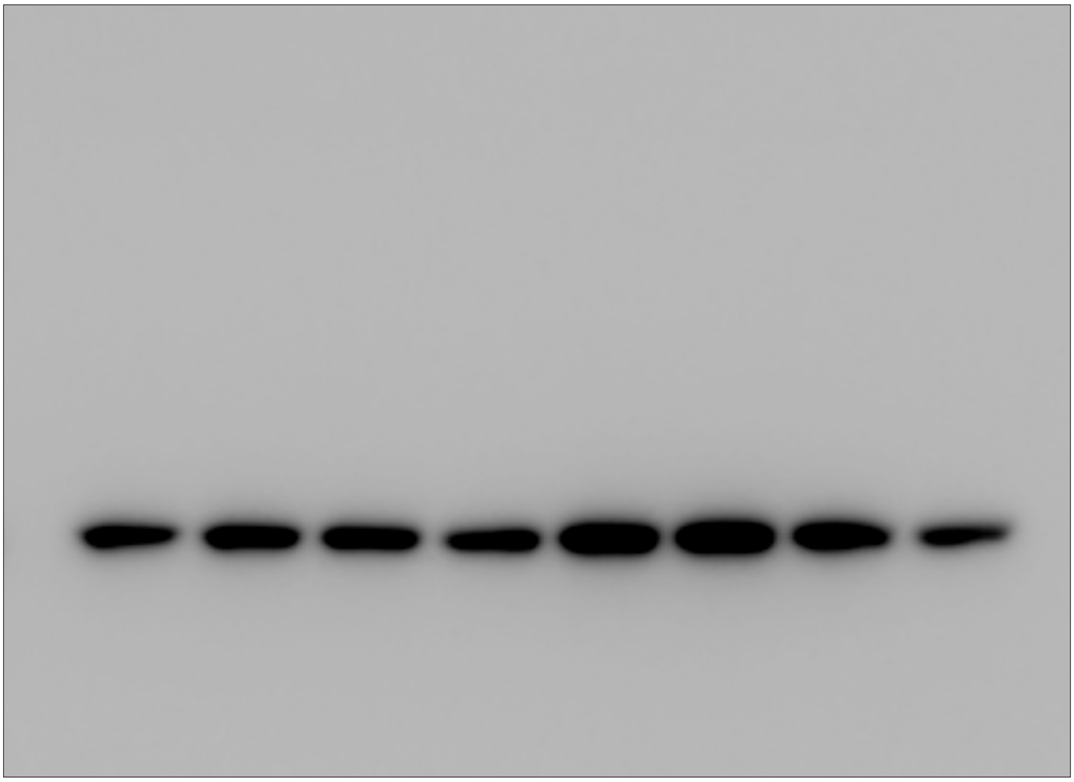

Supplementary figure S9. Raw images in Figure 5b before cropping. (**a**) PML1. (**b**) β-actin.
